# Late radiation necrosis following stereotactic radiosurgery after COVID-19 vaccination: a case report and hypothesis of immune-mediated inflammatory activation

**DOI:** 10.3389/fonc.2026.1895828

**Published:** 2026-07-14

**Authors:** Tugce Kutuk, Marshall Harrell, Kari Flaute, Christina Flores, Neha Rao, Regan Memmott, James Bradley Elder, Joshua Palmer

**Affiliations:** 1 Department of Radiation Oncology, The Ohio State University Wexner Medical Center, Columbus, OH, United States; 2 Division of Medical Oncology, Department of Internal Medicine, The Ohio State University Wexner Medical Center, Columbus, OH, United States; 3 Department of Neurological Surgery, The Ohio State University Wexner Medical Center, Columbus, OH, United States

**Keywords:** brain metastases, COVID-19 vaccination, fluciclovine PET, radiation necrosis, stereotactic radiosurgery

## Abstract

**Background:**

Radiation necrosis (RN) is a recognized complication of stereotactic radiosurgery (SRS) for brain metastases, typically occurring within 6–24 months after treatment. Late RN occurring several years after SRS remains uncommon and may present substantial diagnostic challenges, particularly in patients with prolonged systemic immune exposure and evolving imaging abnormalities.

**Case presentation:**

A 66-year-old female with metastatic non-small cell lung cancer (NSCLC) presented in 2018 with four brain metastases treated with single-fraction SRS to 20 Gy. She subsequently received carboplatin, pemetrexed, and prolonged pembrolizumab therapy with complete extracranial response. Serial surveillance MRIs demonstrated excellent intracranial response and long-term stability for several years. Approximately 46 months after SRS, new enhancing lesions with surrounding edema and hemorrhagic changes developed within previously treated regions. Over subsequent follow ups, imaging demonstrated fluctuating enhancement, progressive FLAIR abnormalities, and mixed interval changes despite predominantly decreased perfusion characteristics suggestive of treatment effect. Fluciclovine positron emission tomography (PET) demonstrated multiple radiotracer-avid lesions concerning recurrent metastatic disease. Due to persistent concern for progression, the dominant right temporal lesion underwent preoperative re-irradiation followed by surgical resection. Histopathology demonstrated RN without viable tumor. During the interval preceding radiographic progression, the patient had also received multiple COVID-19 vaccinations. Although causality cannot be established, systemic immune activation within chronically irradiated tissue may have contributed to inflammatory activation of subclinical radiation injury.

**Conclusion:**

This case highlights the potential for delayed RN several years after SRS and demonstrates the diagnostic limitations of advanced imaging modalities, including amino acid PET tracers, in distinguishing RN from recurrent metastases. Chronic immune priming, prior immunotherapy exposure, and systemic inflammatory activation may contribute to delayed manifestation of RN in susceptible irradiated CNS tissue.

## Introduction

Brain metastases are among the most common intracranial neoplasms and are increasingly managed with stereotactic radiosurgery (SRS), which provides excellent local control while minimizing the neurocognitive toxicity associated with whole-brain radiotherapy (1). However, radiation necrosis (RN) remains a clinically significant delayed toxicity, affecting approximately 5%-25% of patients treated with SRS for brain metastases (2). RN most commonly occurs within 6–24 months following treatment, with a median onset of approximately 7–11 months (3, 4). Although late presentations beyond 18 months have been described, delayed RN occurring several years after SRS remains uncommon and incompletely characterized (5). The increasing use of immune checkpoint inhibitors (ICIs) has introduced additional complexity in distinguishing recurrent tumors from treatment-related effects. Immunotherapy may potentiate inflammatory responses within previously irradiated tissue, potentially increasing the risk of RN (6, 7). Furthermore, advanced imaging modalities, including amino acid positron emission tomography (PET) tracers, while promising, may demonstrate uptake in both recurrent tumor and inflammatory lesions, limiting diagnostic specificity (8, 9).

Emerging evidence suggests that chronically irradiated CNS tissue may remain susceptible to secondary inflammatory or immune-mediated activation long after radiation exposure. Late radiation effects in the CNS are sustained by chronic microglial activation, senescence-associated secretory phenotype (SASP)-mediated cytokine release, and blood-brain barrier disruption that permits ongoing peripheral immune cell infiltration (10). Systemic immune stimulation, including vaccination, may theoretically contribute to inflammatory reactivation within this chronically primed tissue; COVID-19 vaccines have been identified as triggers for radiation recall reactions and have been temporally associated with CNS inflammatory disorders, while mRNA vaccines induce trained immunity signatures characterized by sustained upregulation of the same pro-inflammatory mediators that drive neuroinflammation in irradiated brain tissue (11–14). Although this relationship remains poorly understood and causality has not been established, these overlapping pathways provide biological plausibility for the hypothesis that systemic immune activation could reactivate subclinical radiation injury in the CNS.

We present a case of pathology-proven delayed radiation necrosis developing approximately 46 months after SRS for brain metastases in a patient with prolonged pembrolizumab exposure and repeated COVID-19 vaccination. The case highlights the diagnostic challenges associated with delayed RN, including false-positive fluciclovine PET findings, and raises the hypothesis that systemic immune activation may contribute to delayed inflammatory manifestation of subclinical radiation injury.

## Case presentation

A 66-year-old female presented in April 2018 with a two-week history of right hand weakness. Magnetic resonance imaging (MRI) of the brain demonstrated four enhancing intracranial lesions involving the posterior right temporoparietal lobe, right occipital lobe, and left precentral gyrus with surrounding vasogenic edema. Systemic staging demonstrated right upper lobe pulmonary lesions with mediastinal adenopathy. Biopsy of the lung lesion confirmed adenocarcinoma of pulmonary origin without targetable mutations and with PD-L1 expression of 1%. The patient underwent single-fraction SRS to four intracranial metastases in 2018, receiving 20 Gy in 1 fraction. Treatment was well tolerated. She subsequently received systemic therapy with carboplatin, pemetrexed, and pembrolizumab followed by maintenance pembrolizumab-based therapy through April 2020, achieving complete extracranial response without evidence of systemic progression. Early post-treatment brain perfusion MRI examinations demonstrated near-complete intracranial response with progressive decrease and eventual resolution of the enhancing lesions and associated vasogenic edema. Surveillance imaging remained stable for several years following treatment.

**Figure 1:**
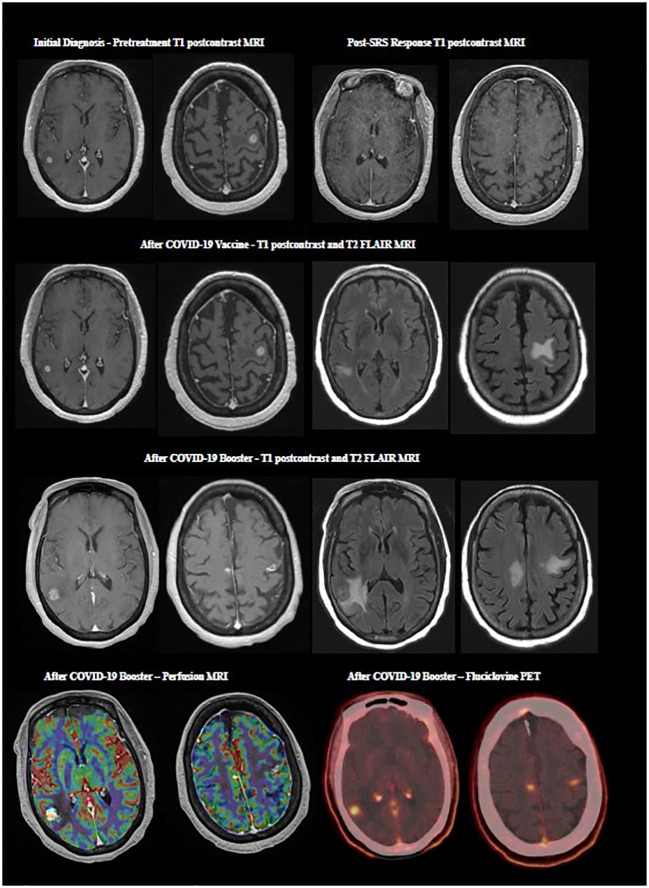
Longitudinal multimodal imaging evolution following stereotactic radiosurgery (SRS) for brain metastases. Pretreatment T1 postcontrast MRI demonstrated multiple enhancing intracranial metastases at initial diagnosis. Early post-SRS MRI showed near-complete intracranial response with minimal residual enhancement. Following COVID-19 vaccination and subsequent booster vaccinations, serial T1 postcontrast and T2 FLAIR MRI examinations demonstrated progressive enhancement, evolving edema, and treatment-related changes. Perfusion MRI demonstrated persistently decreased perfusion favoring radiation necrosis,while subsequent fluciclovine PET demonstrated hypermetabolic uptake concerning for recurrent di sease.

Approximately 46 months after SRS, despite remaining neurologically stable without evidence of extracranial disease progression, brain MRI demonstrated new enhancing lesions involving the left precentral gyrus, right frontoparietal region, and posterior right temporal lobe associated with surrounding edema and hemorrhagic features. Dynamic susceptibility contrast perfusion imaging demonstrated predominantly decreased relative cerebral blood volume, favoring treatment-related change rather than recurrent metastatic disease. Conservative management with pentoxifylline, vitamin E, turmeric, and boswellia was initiated. The patient tolerated conservative therapy without significant adverse effects; however, serial surveillance imaging demonstrated continued radiographic evolution, prompting further diagnostic evaluation. Subsequent serial MRI examinations demonstrated fluctuating lesion size, evolving FLAIR hyperintensity, internal hemorrhagic changes, and mixed interval progression over several years. Despite intermittent mild enlargement of multiple lesions, perfusion characteristics repeatedly favored RN over tumor recurrence. Fluciclovine PET was performed and demonstrated multiple radiotracer-avid lesions, including dominant uptake within the right temporo-occipital lesion (SUVmax 6.1), raising significant concern for recurrent metastatic disease. Serial MRI examinations demonstrated fluctuating enhancement, evolving edema, and persistent low perfusion favoring treatment effect/radiation necrosis over time (**Figure 1**). Given persistent diagnostic uncertainty and continued radiographic progression, the dominant right temporal lesion underwent preoperative hypofractionated radiotherapy to 24 Gy in 3 fractions followed by surgical resection in 2025. Histopathologic evaluation demonstrated RN without viable malignancy, confirming delayed RN. Following surgical resection, the patient experienced an uncomplicated postoperative recovery and remained neurologically stable. Follow-up surveillance MRI demonstrated expected postoperative changes without evidence of recurrent intracranial malignancy, and she continues to undergo routine clinical and radiographic surveillance.

**Figure 2:**
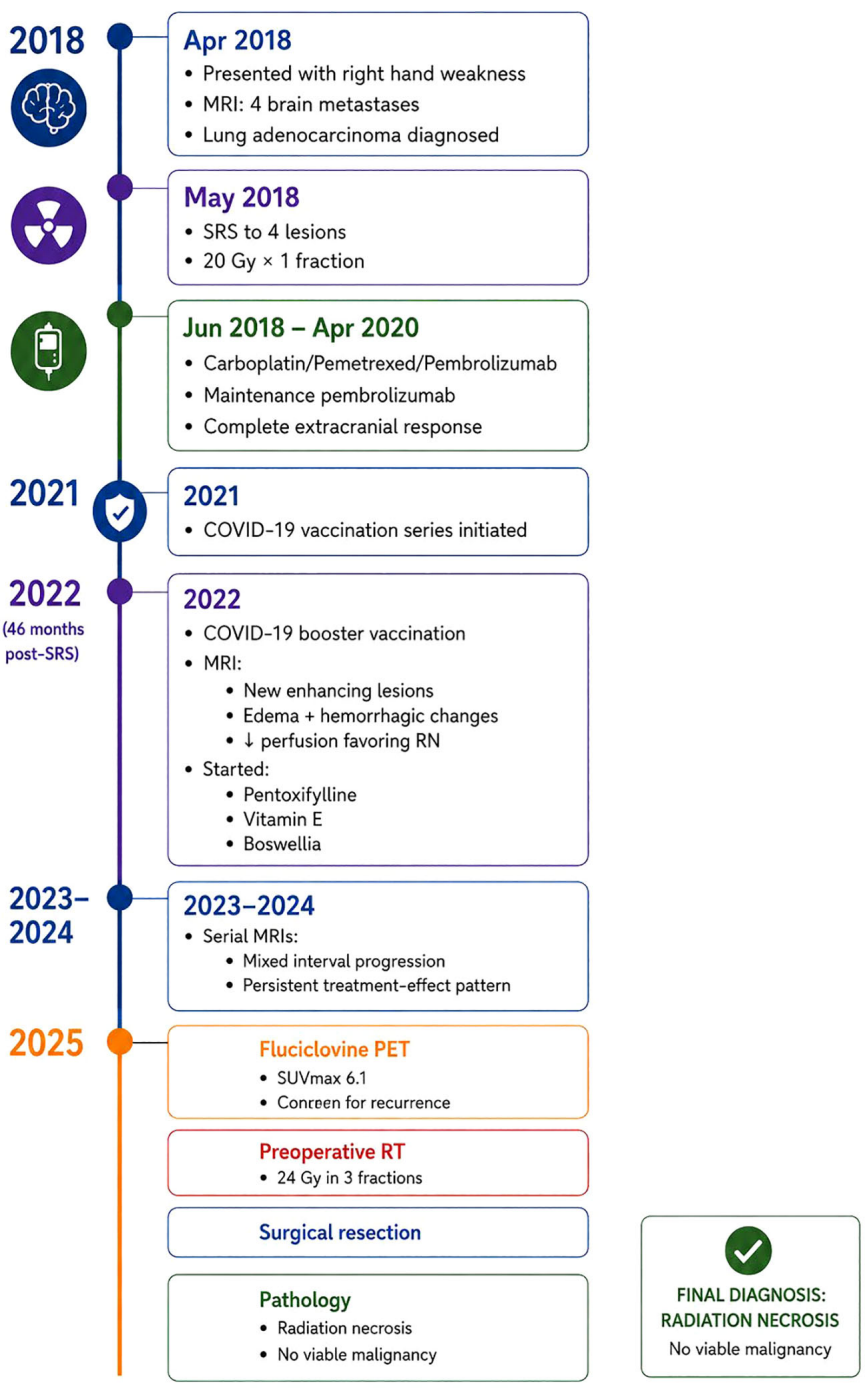
Clinical timeline demonstrating delayed radiation necrosis following SRS and temporal association with COVID-19 vaccination.

Notably, the patient received multiple COVID-19 vaccinations during the time radiographic progression, with serial surveillance brain MRI examinations performed approximately every 3–6 months. The COVID-19 primary vaccination series was initiated in 2021, followed by a booster vaccination in 2022, during which serial MRI examinations documented the emergence and subsequent evolution of the treated lesions. During this period, she also developed transient inflammatory musculoskeletal symptoms, including bilateral hand arthralgias predominantly involving the metacarpophalangeal and proximal interphalangeal joints, with greatest severity in the thumbs, as well as episodic right upper extremity and shoulder pain associated with muscle spasms. She was evaluated by her primary care physician and treated with a methylprednisolone dose pack for presumed inflammatory arthralgia, with symptomatic improvement. Although causality cannot be established, the temporal association raises the possibility that systemic immune activation may have contributed to inflammatory activation within chronically irradiated CNS tissue. The patient’s longitudinal clinical course, imaging evolution, and treatment interventions are summarized in **Figure 2**.

## Discussion

This case demonstrates an unusual pattern of delayed, chronically evolving RN following SRS for brain metastases. Although the patient initially achieved durable intracranial and extracranial disease control, new enhancing lesions developed approximately 46 months after treatment and subsequently demonstrated fluctuating radiographic evolution over several years before pathology ultimately confirmed RN without viable tumor. The prolonged interval between SRS and radiographic progression, combined with persistent imaging abnormalities and false-positive fluciclovine PET uptake, created substantial diagnostic uncertainty throughout the clinical course.

Rather than demonstrating the steadily progressive enhancement typically expected with recurrent metastatic disease, the lesions exhibited a dynamic and indolent radiographic pattern characterized by waxing and waning enhancement, evolving FLAIR abnormalities, intermittent hemorrhagic change, and persistently decreased perfusion characteristics over several years. This fluctuating course is consistent with the current understanding of RN pathophysiology as a self-sustaining feedback loop involving vascular injury, ischemia, neuroinflammation, and perivascular fibrosis (2). Irradiated CNS tissue may persist in a prolonged subclinical inflammatory state, with senescent astrocytes and chronically activated microglia perpetuating cytokine-mediated neuroinflammation through TNF-α, IL-1β, IL-6, and CCL2 signaling pathways (10, 15).

An additional hypothesis-generating feature of this case was the temporal association between repeated COVID-19 vaccination and subsequent radiographic progression. Although causality cannot be established, radiation recall reactions triggered by COVID-19 vaccines have been documented across multiple organ systems. In the WHO pharmacovigilance database VigiBase, the mRNA vaccine tozinameran (BNT162b2) was the most frequently reported non-anticancer agent associated with radiation recall reactions (16). The earliest reports of COVID-19 vaccine-associated radiation recall described acute inflammatory skin reactions developing within days of the second vaccine dose and confined precisely to previously irradiated treatment fields (11). While radiation recall phenomena within the CNS remain poorly characterized, the concept of trained immunity provides a biologically plausible mechanistic framework for persistent inflammatory activation within chronically injured irradiated tissue. mRNA-1273 vaccination has been shown to induce sustained increases in TNF-α and IL-6 production persisting for up to 180 days, in addition to upregulation of CCL2, a key chemokine involved in peripheral immune cell recruitment into irradiated CNS tissue (12). Similarly, BNT162b2 vaccination has been associated with development of a stimulus- and cytokine-dependent innate immune memory phenotype that became evident following booster administration and persisted for at least eight months thereafter (17). These durable alterations in innate immune responsiveness overlap substantially with the inflammatory signaling pathways implicated in chronic radiation-induced neuroinflammation, including microglial activation, endothelial dysfunction, blood-brain barrier disruption, and cytokine-mediated tissue injury. Collectively, these observations provide biologic plausibility for the hypothesis that vaccination-induced systemic immune activation may reactivate or amplify subclinical chronic radiation injury within previously irradiated brain tissue.

Several aspects of this patient’s clinical course further support the possibility that systemic immune activation may have contributed to the evolution of delayed RN. The patient demonstrated prolonged radiographic stability for several years following SRS before developing progressive inflammatory imaging changes during the interval of repeated COVID-19 vaccination. Importantly, the lesions demonstrated a fluctuating radiographic pattern characterized by waxing and waning enhancement, evolving edema, and intermittent hemorrhagic change rather than the continuous monotonic progression typically expected for recurrent metastatic disease. Serial perfusion imaging repeatedly favored treatment-related inflammatory change, and histopathology ultimately confirmed necrosis without viable tumor. Notably, COVID-19 vaccination has independently been associated with rare *de novo* CNS inflammatory disorders, including autoimmune encephalitis, transverse myelitis, and acute disseminated encephalomyelitis, with reported median symptom onset approximately 14 days following vaccination and CSF cytokine analyses demonstrating elevated CXCL-10 (IP-10), indicative of robust T-cell activation and CNS immune recruitment (13). The largest single-center observational cohort of acute CNS inflammation following COVID-19 vaccination identified 38 patients presenting within a median of 15 days after vaccination, including cases of tumefactive demyelination and new-onset demyelinating disease, supporting the capacity of mRNA vaccines to trigger clinically significant CNS-directed immune responses even in previously healthy individuals (18). Furthermore, a recently published review concluded that although the absolute risk remains exceedingly low, COVID 19 vaccination may rarely trigger relapse or exacerbate the initial presentation of inflammatory neurologic disorders in predisposed individuals, an observation particularly relevant to patients harboring chronically irradiated CNS tissue already primed for immune mediated inflammatory activation (19). Additionally, anti-idiotype antibodies directed against ACE2, a protein expressed on neurons and cerebral endothelial cells, have been detected following both SARS-CoV-2 infection and vaccination and have been hypothesized to contribute to endothelial immune activation and vascular dysfunction (20). While these observations remain insufficient to establish a causal relationship, the temporal association, inflammatory radiographic phenotype, documented ability of COVID-19 vaccines to induce CNS-directed immune responses, and mechanistic convergence between vaccine-associated immune activation and chronic radiation injury collectively raise the possibility that vaccination-associated immune stimulation contributed to the transition from subclinical chronic radiation injury to clinically apparent RN.

This case additionally highlights important limitations of advanced imaging in distinguishing RN from recurrent disease. Despite serial perfusion MRI findings favoring treatment effect, fluciclovine PET demonstrated avid lesions with SUVmax of 6.1, exceeding the threshold of 4.8 established by the PURSUE trial as suspicious for recurrence (sensitivity 80%, specificity 85%) (8). Similar diagnostic discordance between advanced MRI, amino acid PET imaging, and histopathology has also been reported in a recent case report (21). The ACR Appropriateness Criteria note that amino acid tracer activity may occur in nonneoplastic conditions including inflammatory processes, limiting specificity in complex post-treatment lesions (9). A meta-analysis of amino acid and FDG-PET reported pooled sensitivity and specificity of 85% and 88%, respectively, for differentiating brain metastasis recurrence from RN (22). The false-positive PET findings in this case underscore that inflammatory RN may demonstrate substantial amino acid tracer uptake, and histopathologic confirmation remains essential when diagnostic uncertainty persists despite multimodality imaging.

As survival improves for patients treated with SRS and modern systemic therapies, clinicians will increasingly encounter delayed and atypical treatment-related effects. This case underscores the importance of integrating longitudinal imaging evolution, advanced imaging interpretation, treatment history, and clinical context when evaluating late enhancing lesions after SRS. Furthermore, the potential interaction between chronic radiation injury, immunotherapy exposure, and systemic immune activation warrants further investigation in long-term survivors.

## Conclusion

This case demonstrates pathology-confirmed delayed evolving RN following SRS for brain metastases, with radiographic progression occurring years after treatment and imaging findings mimicking recurrent metastatic disease. The case highlights the complex interplay between chronic radiation injury, prolonged immune checkpoint inhibitor exposure, systemic immune activation, and advanced imaging interpretation. While causality cannot be established, repeated systemic immune stimulation from COVID-19 vaccination may theoretically contribute to inflammatory activation within chronically irradiated CNS tissue. Histopathologic confirmation remains the gold standard when diagnostic uncertainty persists.

## Patient perspective

The patient was initially encouraged by her excellent response to treatment and the prolonged period of disease stability following SRS and systemic therapy. The development of new enhancing brain lesions several years later was concerning because of the possibility of recurrent metastatic disease despite otherwise excellent cancer control. She described the prolonged period of diagnostic uncertainty, multiple imaging evaluations, and ultimately surgery as emotionally challenging because the possibility of cancer recurrence remained unresolved. Following pathologic confirmation of RN without viable tumor, she expressed relief that the lesions did not represent recurrent cancer. The patient was supportive of sharing her clinical course to help improve understanding of delayed radiation necrosis, increase awareness of its diagnostic challenges, and potentially help other patients facing similar uncertainty.

## Data Availability

The data analyzed in this study is subject to the following licenses/restrictions: This is a case study. Requests to access these datasets should be directed to tugce.kutuk@osumc.edu.
