# Supplementary material for: Late radiation necrosis following stereotactic radiosurgery after COVID-19 vaccination: a case report and hypothesis of immune-mediated inflammatory activation
Source: Front Oncol. 2026 Jul 14;16:1895828. doi: 10.3389/fonc.2026.1895828 (PMC13407089; doi:10.3389/fonc.2026.1895828)
Supplement: Supplementary file 1 [file DataSheet1.pdf]

CARE case report guidelines

CARE Checklist of information to include when writing a case report

(cc) BY-NC-ND

Topic	Item	Checklist item description	Reported on Line
**Title**	1	The diagnosis or intervention of primary focus followed by the words "case report"	Title page
**Key Words**	2	2 to 5 key words that identify diagnoses or interventions in this case report, including "case report"	Title page
**Abstract (no references)**	3a	Introduction: What is unique about this case and what does it add to the scientific literature?	Abstract, Background
	3b	Main symptoms and/or important clinical findings	Abstract, Case Presentation
	3c	The main diagnoses, therapeutic interventions, and outcomes	Abstract, Case Presentation
	3d	Conclusion—What is the main "take-away" lesson(s) from this case?	Abstract, Conclusion
**Introduction**	4	One or two paragraphs summarizing why this case is unique (**may include references**)	Introduction
**Patient Information**	5a	De-identified patient specific information	Introduction
	5b	Primary concerns and symptoms of the patient	Case Presentation, Paragraph 1
	5c	Medical, family, and psycho-social history including relevant genetic information	Case Presentation, Paragraph 1
	5d	Relevant past interventions with outcomes	Case Presentation, Paragraph 1
**Clinical Findings**	6	Describe significant physical examination (PE) and important clinical findings	Case Presentation, Paragraphs 1 and 3
**Timeline**	7	Historical and current information from this episode of care organized as a timeline	Figure 2
**Diagnostic Assessment**	8a	Diagnostic testing (such as PE, laboratory testing, imaging, surveys).	Case Presentation, Paragraphs 1-2
	8b	Diagnostic challenges (such as access to testing, financial, or cultural)	Discussion, Paragraphs 1 and 6
**Therapeutic Intervention**	8c	Diagnosis (including other diagnoses considered)	Case Presentation, Paragraph 2
	8d	Prognosis (such as staging in oncology) where applicable	Case Presentation, Paragraph 1
**Follow-up and Outcomes**	9a	Types of therapeutic intervention (such as pharmacologic, surgical, preventive, self-care)	Case Presentation, Paragraphs 1-2
	9b	Administration of therapeutic intervention (such as dosage, strength, duration)	Case Presentation, Paragraphs 1-2
	9c	Changes in therapeutic intervention (with rationale)	Case Presentation, Paragraph 2
	10a	Clinician and patient-assessed outcomes (if available)	Case Presentation, Paragraphs 2-3

10b Important follow-up diagnostic and other test results ....

10c Intervention adherence and tolerability (How was this assessed?) .... Case Presentation, Paragraph 2

10d Adverse and unanticipated events .... Case Presentation, Paragraphs 1 and 3

Discussion

11a A scientific discussion of the strengths AND limitations associated with this case report .... Discussion

11b Discussion of the relevant medical literature **with references** .... Discussion

11c The scientific rationale for any conclusions (including assessment of possible causes) .... Discussion

11d The primary "take-away" lessons of this case report (without references) in a one paragraph conclusion .... Discussion

Patient Perspective **12** The patient should share their perspective in one to two paragraphs on the treatment(s) they received .... Patient Perspective section

Informed Consent **13** Did the patient give informed consent? Please provide if requested .... Yes **Yes** ☐ **No** ☐
